# The effects of one-night sleep deprivation and one-night recovery sleep on endurance cycling performance

**DOI:** 10.1007/s00421-025-05908-w

**Published:** 2025-07-25

**Authors:** Chiara Gattoni, Michele Girardi, Amir-Homayoun Javadi, Barry Vincent O’Neill, Samuele Maria Marcora

**Affiliations:** 1 The Lundquist Institute for Biomedical Innovation at Harbor-UCLA Medical Center, Torrance, CA USA; 2 School of Psychology, University of Kent, Canterbury, UK; 3 Unilever Science & Technology, Unilever R&D Colworth Science Park, Sharnbrook, Bedford, UK; 4 Department of Quality of Life Studies, University of Bologna, Rimini, Italy; 5 School of Sport and Exercise Sciences, University of Kent, Canterbury, UK

**Keywords:** EEG, Perception of effort, Sleepiness, Vigilance, Time trial

## Abstract

**Purpose:**

Sleep deprivation (SD) seems to impair endurance performance. However, little research is available on the effects of SD in the context of repeated bouts of endurance exercise. The effect of recovery sleep on endurance performance is also poorly understood.

**Methods:**

Twenty-six male amateur cyclists/triathletes (mean ± SD: age 30.5 ± 8.8 yr, $\dot{\mathrm{V}}{\mathrm{O}}$_2peak_ 55.3 ± 4.9 mL/kg/min) were randomly allocated to SD and CONTROL groups and tested over 3 consecutive days. After baseline testing (day 1), the SD group did not sleep for 25 h (day 2) and slept normally the following night (day 3). The CONTROL group slept normally throughout the experiment. Endurance performance was assessed during 40-min moderate-intensity constant-workload cycling and 20-min time trial. Electroencephalography, questionnaires, and psychomotor vigilance task were used to assess sleepiness and vigilance before cycling. Ratings of perceived exertion (RPE), Feeling scale (FS), heart rate (HR), and blood lactate were assessed during cycling.

**Results:**

Sleepiness and vigilance were impaired after SD (*p* < 0.05), but returned to baseline after recovery sleep. RPE (*p* = 0.023) and FS (*p* = 0.013) assessed during the 40-min cycling were negatively affected by SD, but returned to baseline after recovery sleep. HR and blood lactate were not affected by SD. No significant interaction effect was observed on time-trial work.

**Conclusion:**

In the context of 3 consecutive days of endurance cycling, 25-h SD negatively affected moderate-intensity performance through higher RPE, but did not affect the 20-min time trial. One-night recovery sleep was sufficient to restore moderate-intensity endurance cycling performance.

## Introduction

Sleep deprivation (SD) can be defined as “any condition of insufficient sleep, whether acute or chronic, total or partial” (Avidan 2017). It has been extensively demonstrated that SD increases subjective feelings of sleepiness and general fatigue and negatively affects individuals’ mood (Leonard et al. 1998; Belenky et al. 2003). It is also clear that SD has detrimental effects on cognition causing clear deficits in vigilance and sustained attention (Lowe et al. 2017). Decrements in cognitive performance caused by SD have been associated with a decreased activity and function in several specific areas of the brain (Boonstra et al. 2007). Specifically, findings from neuroimaging studies have suggested that the prefrontal cortex seems to play a key role when individuals encounter sleep loss (Thomas et al. 2000; Muzur et al. 2002; Asplund and Chee 2013; Zhu et al. 2018), and electroencephalography (EEG) experiments have observed spectral power shifts toward lower frequencies following SD (Cajochen et al. 1995; Finelli et al. 2000; Boonstra et al. 2007).

While extensive research has been conducted on the effects of SD on neurocognitive function (Lowe et al. 2017), the number of studies investigating the effects of sleep loss on physical performance are still limited (Fullagar et al. 2015; Thun et al. 2015; Lopes et al. 2023). A recent meta-analysis found that endurance performance is negatively affected by acute SD (Lopes et al. 2023). In particular, 25–50 h of continuous wakefulness significantly impaired endurance running, walking, and cycling performance, during both constant-workload time-to-exhaustion testing (Martin 1981; Martin and Chen 1984; Temesi et al. 2013) and time trials (Oliver et al. 2009; Roberts et al. 2019). There is, however, very little research on the effects of SD in the context of bouts of endurance exercise repeated over consecutive days (Gattoni et al. 2022). This is an important area of research because sleep loss often occurs during multi-stage and ultra-endurance events in which athletes perform endurance exercise repeatedly over several days (Kishi et al. 2024). Sleep loss in the context of repeated bouts of physical tasks requiring endurance also occurs in soldiers during military operations (Nindl et al. 2018).

Another open research question is whether one night of recovery sleep can restore endurance performance. Previous studies have shown that recovery sleep following SD is beneficial to increase cognitive performance (Balkin et al. 2005; Wesensten et al. 2005; Drummond et al. 2006; Rupp et al. 2009) and that one night of recovery sleep seems to be sufficient to restore baseline levels after two nights of total SD (Drummond et al. 2006). However, to the best of our knowledge, the effects of acute recovery sleep on physical performance have not been tested as yet. The only data available come from our single case study (Gattoni et al. 2022), in which a prolonged constant-workload running test was repeated over 5 consecutive days during alternating nights of SD and recovery sleep. A visual analysis of the data highlighted perceptual and effort-related improvements following the nights of recovery sleep in the ultra-endurance athlete tested. Nevertheless, these are preliminary findings that need to be corroborated.

The aim of the present study was to investigate the acute effects of one-night SD and the subsequent night of recovery sleep on endurance performance in the context of cycling exercise tasks repeated over 3 consecutive days. We hypothesized that 25 h of SD would impair endurance performance during: (1) a 40-min moderate-intensity constant-workload cycling task, and (2) a 20-min all-out cycling time-trial. In addition, we hypothesized that one subsequent night of recovery sleep would be sufficient to restore endurance cycling performance to rested levels.

## Methods

### Participants

Twenty-six male amateur cyclists and triathletes (means ± SD: age 30.5 ± 8.8 yr, body mass 72 ± 9 kg, height 1.77 ± 0.08 m, peak oxygen uptake ($\dot{\mathrm{V}}{\mathrm{O}}$_2peak_) 55.3 ± 4.9 mL/kg/min) undergoing more than 3 h of moderate- to high-intensity cycling training per week were recruited. Participants were also selected based on their chronotype and sleep quality/disturbances over a 1-month period before the experiment. Chronotype and quality of sleep were assessed using the Horne and Ostberg “morningness-eveningness” questionnaire (MEQ) (Horne and Ostberg 1976) and the Pittsburgh Sleep Quality Index (PSQI) (Buysse et al. 1989), respectively. Only moderate-evening/intermediate/moderate-morning types (MEQ score between 30 and 70) and good sleepers (PSQI < 5) were included in the study. Participants with any pulmonary, cardiovascular or metabolic disease and those unable to perform the required physical tasks were excluded. Prior to participation, all participants were informed about the study protocol and signed an informed consent. All the procedures used were approved by the School of Sports and Exercise Sciences Ethics Committee at the University of Kent (ID: 13,220,152,016) and conducted in conformity with the Declaration of Helsinki.

### Experimental design

A randomized, parallel control-group design was used. After baseline testing in normal sleep conditions, participants were randomly allocated to two independent groups. The experimental group (means ± SD: age 31.1 ± 9.6 yr, body mass 74 ± 10 kg, height 1.75 ± 0.07 m, $\dot{\mathrm{V}}{\mathrm{O}}$_2peak_ 54.2 ± 5.1 mL/kg/min) was instructed to undergo one night of SD (i.e., 25-h SD) and recover the following night (recovery sleep) (experimental treatment). The control group (means ± SD: age 29.8 ± 9.3 yr, body mass 69 ± 7 kg, height 1.75 ± 0.10 m, $\dot{\mathrm{V}}{\mathrm{O}}$_2peak_ 56.4 ± 4.6 mL/kg/min) was asked to maintain normal sleep patterns throughout the entire experiment (control treatment). The experiment was conducted in the Psychobiology Laboratory of the School of Sport and Exercise Sciences, University of Kent. The night of SD took place in a quiet room of the same building.

Participants attended the laboratory on four separate occasions (one *Familiarization* and three *Experimental visits*). The *Familiarization* was carried out between 3–6 days prior to the experimental visits. During this visit, participants performed a ramp incremental cycling test and were familiarized with all the experimental testing and procedures. The *Experimental visits* were conducted in 3 consecutive days, after one night of normal sleep (day 1, baseline), one night of SD (SD group) or normal sleep (CON group) (day 2, SD condition), and one night of recovery sleep (SD group) or normal sleep (CON group) (day 3, recovery sleep condition). All visits commenced at 8:00 am and were conducted between 8:00 and 11:00 am, a time of day during which the negative impact of lack of sleep on alertness is most pronounced (Mollicone et al. 2010). During each experimental visit, participants performed the same testing (see *Testing procedures* and *Experimental visits*). All physical tests were performed on an electromagnetically braked cycle ergometer (Cyclus2, RBM elektronik-automation GmbH, Leipzig, Germany). The seat and handlebar positions were adjusted for each participant during the familiarization visit and maintained constant throughout the entire experiment.

Participants were given a standardized light breakfast before each experimental visit and were instructed not to eat in the 12 h preceding the visits. Participants were also asked to maintain their habitual diet and drink an amount of water equal to 35 mL/kg of body mass per day. Caffeine and alcohol were prohibited in the 12 h before each visit. Participants maintained their everyday activities throughout the entire experiment but avoided any additional strenuous physical activity in the 24 h preceding each visit. To monitor dietary, sleep and physical activity behavior, participants completed a food, sleep and physical activity diary during the experiment. A monetary reward (i.e., £100 in Amazon voucher) was provided to participants upon completion of the experiment.

### Testing procedures

#### Familiarization

Participants underwent an initial health screening consisting of a PAR-Q questionnaire. They were also required to inform the researchers about any acute illness, infection and/or injury and to give notice of any medication/drug taken. Following the collection of the main anthropometric measures, participants performed a ramp incremental cycling test. The test commenced immediately after a 5-min warm-up at 100 W, and consisted of work rate increments of 30 W/min (i.e., 1 W every 2 s) until exhaustion, defined as a drop of cadence below 60 rpm despite strong verbal encouragement. Pulmonary gas exchange measurements were collected breath-by-breath throughout the entire test (MetaLyzer 3B, Cortex Biophysik GmbH, Leipzig, Germany). Finger capillary blood samples (i.e., 10 µl) were taken before and 60 s after the test to measure blood lactate concentration (Biosen; EFK Diagnostics, London, UK) (Howley et al. 1995). Heart rate (HR) was collected throughout the entire test (Polar V800, Polar Electro Oy, Kempele, Finland). Ratings of perceived exertion (RPE) were recorded every minute using the Borg’s 15-point scale (Borg 1998). Participants received standard instructions about the scale prior to testing. Moreover, anchoring procedures for the determination of low and high anchor points in the scale were used during the incremental test (Noble and Robertson 1996). Participants were then familiarized with the testing protocols used in the *Experimental visits*.

#### Experimental visits

The testing procedures were identical in each visit. To verify that participants followed all the instructions provided during the *Familiarization* visit, they completed a pre-experimental checklist. At this point, a standardized breakfast consisting of one glass of orange juice (150 mL, 32 kcal, Tropicana Trop 50, Tropicana Products, Chicago, Illinois, USA) and two energy bars (90 kcal per bar, Special K, Kellogg Company, Battle Creek, Michigan, USA) was provided.

To assess subjective levels of sleepiness and mood, participants completed the Karolinska Sleepiness Scale (Åkerstedt and Gillberg 1990) and the Brunel Mood Scale (BRUMS), respectively (Terry et al. 2003) (see *Psychological questionnaires*). Prior to testing, participants wore an electroencephalography (EEG) cap (see *EEG procedures*) and completed a 12-min Alpha Attenuation Test (AAT), a valid and sensitive EEG test to objectively assess sleepiness (Stampi et al. 1995) (see *AAT*). Subsequently, they performed a 10-min psychomotor vigilance task (PVT), a test used to objectively measure vigilance impairments induced by sleep loss (Basner and Dinges 2011) (see *PVT*).

After a 3-min warm-up at their preferred intensity, and the completion of a motivation questionnaire (see *Psychological questionnaires*), participants proceeded to the cycling testing, comprising two parts: (1) a 40-min constant-workload cycling at 60% of participants’ $\dot{\mathrm{V}}{\mathrm{O}}$_2peak_; and (2) a 20-min all-out time trial. The two cycling bouts were separated by 5-min resting. Prior to the 20-min time trial, participants were instructed to give their best performance and to do as much work as possible. Participants did not receive any verbal encouragement during the time trial to avoid experimenter bias. They were allowed to see the time left during the time trial, but they were not aware of their performance throughout the entire experiment. To ensure maximal effort in all three time trials, monetary rewards of £300, £200, and £100 in Amazon vouchers were awarded to the participants with the first, second, and third-best average time-trial performance in each group.

Capillary blood samples were collected from participants’ fingertips immediately before the test, at 8-min intervals during the 40-min constant-workload cycling and 60 s after the 20-min time trial. Blood lactate concentrations were then measured. RPE and ratings of pleasure/displeasure (affect) were collected every 5 min throughout the entire test using the Borg’s 15-point scale (Borg 1998) and the bipolar Feeling scale (FS) (Hardy and Rejeski 1989), respectively. HR was collected continuously using the Polar monitor.

To estimate the workload perceived, participants completed the NASA-TLX multidimensional scale (Hart and Staveland 1988) after the 20-min time trial. To assess changes in sleepiness and mood state, participants also completed the Karolinska Sleepiness Scale and BRUMS (see *Psychological questionnaires*).

### Sleep procedures

All participants slept normally the night before the first experimental visit (day 1). To minimize sleep inertia effects (Tassi and Muzet 2000), they woke up at around 7:00 am and arrived at the laboratory by 8:00 am. After the first visit, the SD group was instructed to abstain from sleep for 25 h, while the CON group was instructed to maintain normal sleep at home the night before day 2. Participants in the SD group spent the night of SD (from 8:00 pm to 8:00 am) in the School of Sport and Exercise Sciences student hub. To prevent napping, food consumption, and the use of caffeinated beverages, participants were closely monitored by a research staff member. Throughout the night, participants engaged in non-cognitively demanding activities, such as reading magazines, watching movies and documentaries, playing video games, and having a conversation with the researcher (Gattoni et al. 2021; Gattoni et al. 2022; Marcora et al. 2009). At 2:00 am, participants were allowed to eat one energy bar (90 kcal, Special K, Kellogg Company, Battle Creek, Michigan, USA). The following morning (day 2), participants attended the second visit and were instructed to sleep at home that night and return for the third and final visit the morning after (day 3). Participants in the SD group were allowed to go to sleep earlier (from 9:00 pm) compared to the CON group (between 10:00 and 11:00 pm). Throughout the 3 experimental days, all participants were strictly advised against taking any naps. To monitor and quantify their sleep–wake activity, all participants wore a wrist actigraph device (AW Spectrum PRO, Philips Respironics, Murrysville, Pennsylvania, USA) for the entire duration of the experiment, starting from 8:00 am the day before the study began.

### EEG procedures

EEG data were collected using a wireless 32-channel cap (g.Nautilus EEG system, g.tec Medical Engineering GmbH, Schiedlberg, Austria) with an active, gel-based Ag/AgCl electrode technology (g.LADYbird; g.tec Medical Engineering GmbH, Schiedlberg, Austria). The 10–20 international system was used for the electrode placement in the cap, with the reference on the right earlobe and the ground electrode on the AFz site. Sampling frequency was set up at 500 Hz with 12-bit resolution. A 48–52 Hz notch filter was also applied. Electrode impedance was checked prior to testing and maintained below 30 kΩ. EEG data were wirelessly transmitted through a receiver connected to the laboratory computer. Data acquisition and analysis were performed using MATLAB software (R2014a, MathWorks, Natick, Massachusetts, USA).

### Alpha Attenuation Test

The AAT was used to neurophysiologically assess alertness decrements induced by SD (Stampi et al. 1995). Participants were asked to sit in a normally lit laboratory (100–150 lx), remain relaxed but awake (Stampi et al. 1995) while looking at a fixation point (i.e., a white asterisk against a black background) displayed on a computer screen in front of them (López Zunini et al. 2013). The test consisted of alternating eyes closed and eyes opened every 2 min for a total duration of 12 min, while EEG data were continuously recorded. To minimize noise in the EEG signal, participants were instructed to refrain from blinking, moving, and contracting any facial muscles.

### EEG data analysis

EEG data from eight participants in the SD group and six participants in the CON group satisfied the predefined quality criteria and were, therefore, retained for analysis. We discarded any dataset in which the Fz electrode, our key channel, showed more than 25% artifacts in any session, usually owing to excessive head movement or sweating that potentially short circuited neighboring electrodes.

The EEG data analysis was conducted at the sensor level using SPM v12 (Statistical Parametric Mapping, Wellcome Trust, London, UK). The data were filtered in the 0.5–30 Hz range using the 7th order Butterworth filter, downsampled to 128 Hz, and montaged based on average electrode activity. Eye blinks were subsequently removed using Fp2 electrode activity. Spatial confounds were identified based on Singular Value Decomposition (SVD) mode, and sensor data were corrected using the Signal-Space Projection (SSP) correction mode. A maximum of two components of spatial confounds was removed from the EEG data.

Our primary focus was on the Fz electrode activity and the low-alpha (9–11 Hz) frequency band. A time–frequency analysis was conducted within the frequency range of 0.5–15 Hz using Morlet wavelet transformation with seven wavelet cycles. Time–frequency data were then rescaled to log-ratio based on the 100 ms activity preceding the start of each data section. Finally, data were averaged over time and frequency bands.

### Psychomotor vigilance task

The computerized version of the 10-min PVT was used to assess SD-induced reductions in vigilance (Basner and Dinges 2011). Participants were instructed to press ‘spacebar’ on the keyboard with their preferred hand upon the appearance of a visual stimulus at the center of the computer screen. They were required to respond as quickly as possible throughout the entire test. The visual stimulus, a fixed graphic bullseye on a white background, appeared with a randomized inter-stimulus interval (i.e., period between a response and the next stimulus) ranging from 2000 to 10,000 ms. Valid reaction times were defined as ≥ 150 ms, with responses < 150 ms considered false starts (errors of commission), and responses ≥ 500 ms counted as lapses (errors of omission). Wrong responses (i.e., pressing another button in the keyboard) and no responses (i.e., > 30,000 ms) were also considered errors. Feedback (i.e., reaction time in ms) was provided after each response, appearing centrally on the screen for 1000 ms before the next stimulus. In case of responses < 150 ms, a “false start” message was displayed. Simple reaction time (RT in ms) and lapses (≥ 500 ms) were used as sensitive measures to SD (Basner and Dinges 2011). The PVT was generated and executed using E-Prime 2.0 software (Psychology Software Tools, Inc, Pennsylvania, USA).

### Psychological questionnaires

*Sleepiness*: The Karolinska Sleepiness Scale (Åkerstedt and Gillberg 1990) was employed to assess subjective levels of sleepiness before the experimental visits. This nine-point Likert scale ranges from 1 (extremely alert) to 9 (very sleepy, great effort to keep awake, fighting sleep).

*Mood*: The BRUMS (Terry et al. 2003) was utilized to assess subjects’ mood before and after the experimental visits. This questionnaire comprises 24 items (e.g., tired, anxious, nervous, confused, energetic, active) to be answered on a 5-point Likert scale (where 0 = not at all, 1 = a little, 2 = moderately, 3 = quite a bit, 4 = extremely). Items are allocated into six specific subscales: anger, confusion, depression, fatigue, tension, and vigor. Each subscale, consisting of four related items, yields a score between 0 and 16.

*Perceived workload*: The NASA-TLX multidimensional scale (Hart and Staveland 1988) was used to assess the subjective workload perceived during the cycling tests. It includes six subscales: mental demand, physical demand, temporal demand, performance, effort and frustration. Participants indicated their experience by circling one of the 20 line intervals present on each subscale, matching their perceived workload. Each subscale includes two endpoint descriptors, with “very low” on the left and “very high” on the right. Notably, the performance-related subscale is reversed, ranging from “good” on the left to “poor” on the right.

*Motivation*: Intrinsic motivation and success on task motivation scales (Matthews et al. 2001) were employed to assess participants’ motivation related to the cycling test. Each scale includes seven items to be scored on a five-point Likert scale (where 0 = not at all, 1 = a little, 2 = somewhat, 3 = very much, 4 = extremely). The total score range for each scale is between 0 and 28.

### Endurance cycling performance

Moderate-intensity endurance cycling performance was measured as time to exhaustion (TTE) predicted using the RPE measured during the 40-min constant-workload test on day 1–3. This approach was selected for different reasons: (1) long-duration endurance cycling performance tests in laboratory settings are difficult to conduct due to boredom and other motivational factors; (2) RPE scales linearly with TTE (Nicolò et al. 2019); (3) conducting two maximal performance tests consecutively was impractical, making necessary the use of this approach. A linear regression analysis was conducted by fitting RPE versus time using a constant intercept: $RPE(t)=a\cdot x(t)+6$; where *a* is the slope of the linear regression, *x* is the time, and 6 is the constant intercept (i.e., RPE at rest). TTE was then predicted by solving the equation: $TTE=(20-6)/a$. This predictive equation is based on the assumption that RPE linearly increases over time during a TTE test and that exhaustion occurs at RPE = 20 (Nicolò et al. 2019). A similar approach has been used in previous studies for $\dot{\mathrm{V}}{\mathrm{O}}$_2peak_ prediction using RPE (Eston et al. 2005; Faulkner et al. 2007).

Total work during the 20-min time trial was used as the measure of high-intensity endurance cycling performance.

### Statistical analysis

The Shapiro–Wilk test, histograms, Q-Q plots, and boxplots were initially employed to check for data normality. A log10 transformation was applied to the predicted TTE data, as a result of not being normally distributed. Two-way mixed-model ANOVAs (group × day) were used to analyze differences in cycling performance (i.e., predicted TTE and time-trial work), vigilance during the PVT (i.e., reaction time and errors number), alpha power during the AAT, sleepiness, mood and motivation. Three-way mixed-model ANOVAs (group × day × time) were used to analyze physiological (i.e., HR and lactate) and perceptual responses (i.e., RPE and FS) during the cycling testing. When the assumption of sphericity was not met, the Greenhouse–Geisser correction was applied. Significant group × day × time interaction effects were followed-up by two-way mixed-model ANOVAs (group × day) at each time point during the cycling testing. Significant group × day interactions were followed-up using one-way repeated-measure ANOVAs within each group. In case of significance, pairwise comparisons were investigated using the Fisher’s LSD method.

Data are presented as means ± SD, unless stated otherwise. Statistical significance was accepted at *p* < 0.05 level. The SPSS package (version 24.0; SPSS, Chicago, Illinois, USA) was used for all statistical analyses.

## Results

**Fig. 1:**
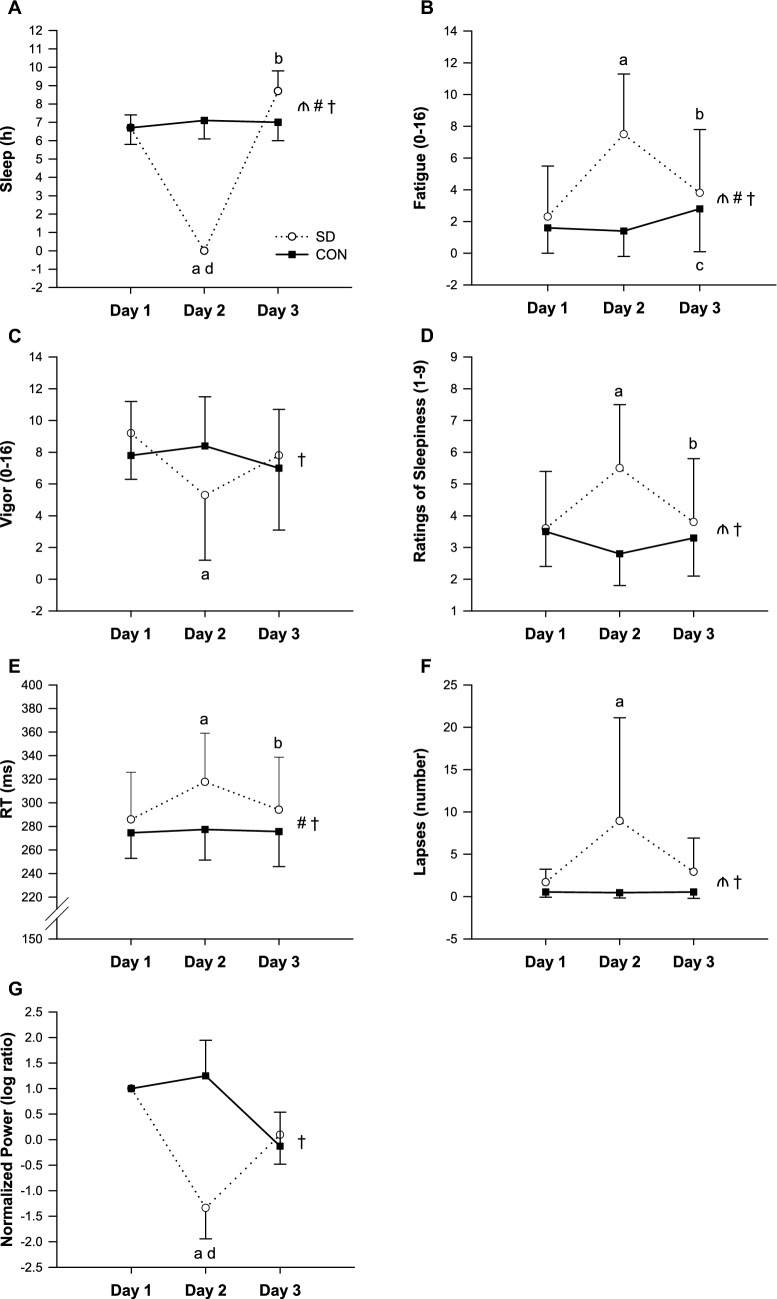
Group mean ± SD changes in the hours of sleep (**A**), subjective levels of fatigue (**B**), vigor (**C**) and sleepiness (**D**), PVT reaction time (RT) (**E**) and number of lapses (**F**) measured on day 1, day 2, and day 3 in the SD group (open line) and CON group (solid line), and group mean ± standard error of the channel Fz mean changes in the normalized alpha power activity during the AAT (**G**) measured on day 1, day 2, and day 3 in the SD group (open line) and CON group (solid line). ₼ = significant main effect of group (*p* < 0.05); # = significant main effect of day (*p* < 0.01); † = significant group × day interaction effect (*p* < 0.05); a = SD group different from day 1 (*p* < 0.05); b = SD group different from day 2 (*p* < 0.05); c = CON group different from day 2 (*p* < 0.05), d = SD group different from CON group (*p* < 0.05)

The duration of sleep per night for each group is presented in Fig. 1A.

### Manipulation checks

#### Subjective sleepiness

Ratings of sleepiness revealed a significant group × day interaction effect of SD (*p* = 0.005) (Fig. 1D). Follow-up analysis indicated higher levels of subjective sleepiness on day 2 compared to day 1 (*p* = 0.012) and subsequent improvements on day 3 compared to day 2 (*p* = 0.045) in the SD group. No significant differences were found between day 1 and day 3 (*p* = 0.712). The one-way ANOVA conducted on the CON group showed no significant simple main effect of day on subjective sleepiness (*p* = 0.310).

#### Alpha Attenuation Test

Before performing the statistical analysis of this objective sleepiness test, the alpha power eyes closed/open on day 2 and day 3 were normalized by day 1. The two-way ANOVA revealed a significant group × day interaction effect (*p* = 0.039). Follow-up analysis showed a significant simple main effect of group at day 2 (*p* = 0.011), but not at day 3 (*p* = 0.401) (Fig. 1G).

#### Mood and perceived workload

Subjective levels of fatigue (Fig. 1B) and vigor (Fig. 1C) assessed using the BRUMS showed significant group × day interaction effects (fatigue, *p* < 0.001; vigor, *p* = 0.038). Notably, the SD group presented greater levels of fatigue (*p* = 0.001) and lower levels of vigor (*p* = 0.005) on day 2 compared to day 1, and lower levels of fatigue (*p* = 0.024) on day 3 compared to day 2. No significant increments in vigor were found on day 3 compared to day 2 (*p* = 0.139). No significant differences were found between day 1 and day 3 (fatigue, *p* = 0.241; vigor, *p* = 0.362). The one-way ANOVAs conducted on the CON group showed a tendency towards significance on fatigue (*p* = 0.065), exhibiting significantly higher levels of fatigue on day 3 compared to day 2 (*p* = 0.030) and no significant simple main effect of day on vigor (*p* = 0.510). The NASA-TLX completed immediately after the cycling testing showed no significant group × day interaction effects.

#### Psychomotor vigilance task

Mean RT during the PVT showed a significant group × day interaction effect for SD (*p* < 0.001) (Fig. 1E). Follow-up analysis revealed significant impairments in the SD group on day 2 (*p* < 0.001) followed by subsequent improvements on day 3 after recovery sleep (*p* = 0.002). No significant differences were observed between day 1 and day 3 (*p* = 0.142). The one-way ANOVA conducted on the CON group showed no significant simple main effect of day on mean RT (*p* = 0.820).

The number of lapses during the PVT (Fig. 1F) also exhibited a significant group × day interaction effect (*p* = 0.045). Specifically, it was significantly higher on day 2 compared to day 1 in the SD group (*p* = 0.049), while no significant differences were observed between day 2 and day 3 (*p* = 0.080). The one-way ANOVA conducted on the CON group showed no significant simple main effect of day on number of lapses (*p* = 0.931).

### Motivation

**Fig. 2:**
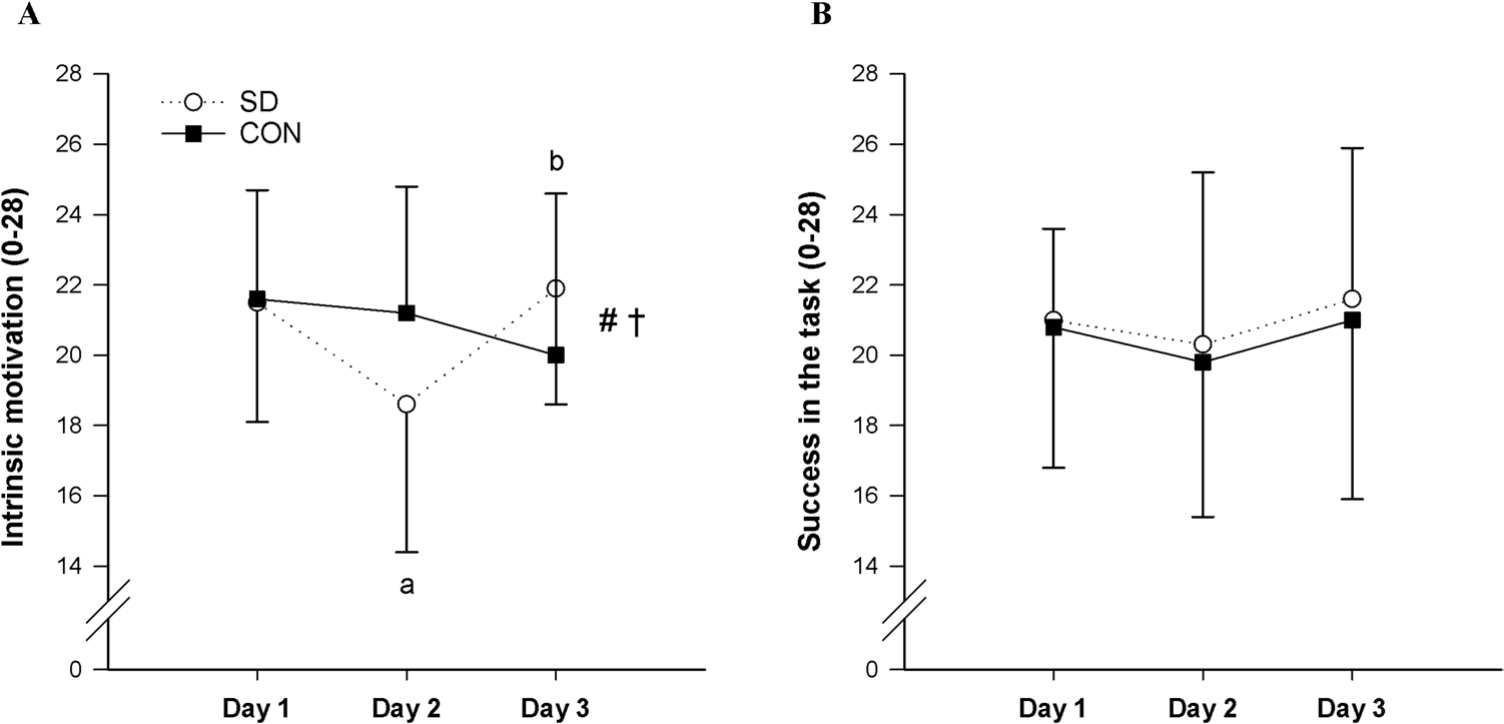
Group mean ± SD changes in intrinsic motivation (**A**) and success in the task motivation (**B**) measured on day 1, day 2, and day 3 in the SD group (open line) and CON group (solid line). # = significant main effect of day (*p* < 0.01); † = significant group × day interaction effect (*p* < 0.001); a = SD group different from day 1 (*p* < 0.001); b = SD group different from day 2 (*p* < 0.01)

A significant group × day interaction effect on intrinsic motivation was found (*p* < 0.001) (Fig. 2A). Follow-up one-way ANOVAs revealed significant lower levels of intrinsic motivation in the SD group on day 2 vs day 1 (*p* < 0.001) and vs day 3 (*p* = 0.001), and no significant differences on day 1 vs day 3 (*p* = 0.635). The one-way ANOVA performed on the CON group showed no significant simple main effect of day on intrinsic motivation (*p* = 0.097). No group × day (*p* = 0.948) and no main effect of day (*p* = 0.128) were found for success motivation (Fig. 2B).

### 40-min constant-workload cycling—perceptual and physiological responses

**Fig. 3:**
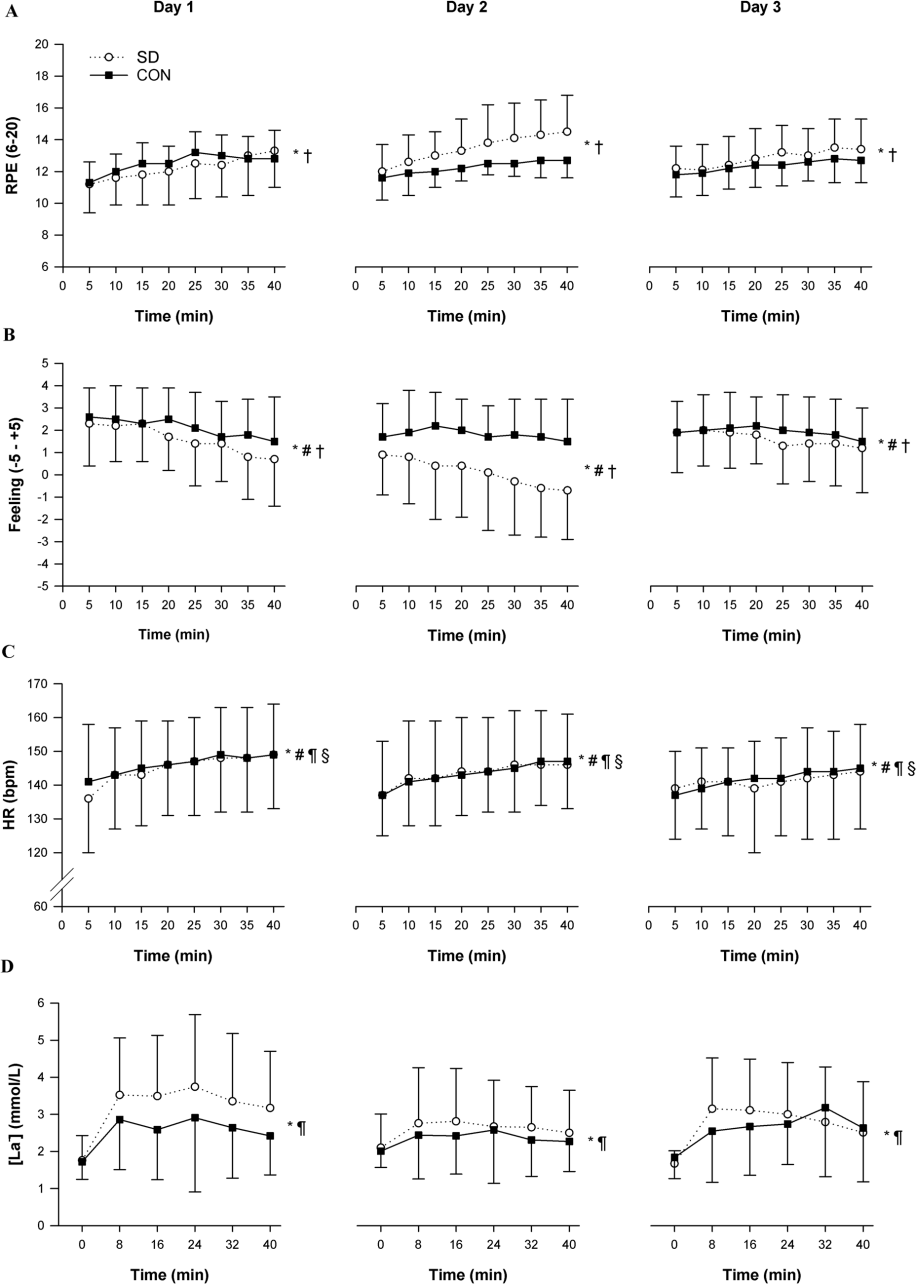
Group mean ± SD changes in RPE (**A**) Feeling scale, (**B**) HR, (**C**) and blood lactate concentrations (D) measured during the 40-min constant-workload cycling on day 1, day 2, and day 3 in the SD group (open line) and CON group (solid line). * = significant main effect of time (*p* < 0.01); # = significant main effect of day (*p* < 0.01); † = significant group × day interaction effect (*p* < 0.05); ¶ = significant day × time effect (*p* < 0.05); § = significant group × day × time interaction effect (*p* < 0.05)

The main effect of time was significant for all variables (RPE, *p* < 0.001; affective valence, *p* = 0.001; HR, *p* < 0.001; blood lactate, *p* = 0.001) (Fig. 3).

No significant group × day × time interaction effect was found for RPE (*p* = 0.118). However, we observed a significant group × day interaction effect (*p* = 0.013) (Fig. 3A). Follow-up one-way ANOVAs revealed a significant simple main effect of day on RPE during the 40-min constant-workload cycling test in the SD group (*p* = 0.025), but not in the CON group (*p* = 0.520). Main pairwise comparisons in the SD group revealed significantly higher RPE on day 2 vs day 1 (*p* = 0.023), and no significant differences between day 3 vs day 2 (*p* = 0.085) and day 1 vs day 3 (*p* = 0.186).

No significant group × day × time interaction effect was found for affective valence (*p* = 0.307). However, a significant group × day interaction effect was observed (*p* = 0.040) (Fig. 3B). Follow-up one-way ANOVAs revealed a significant simple main effect of day on affective valence during the 40-min constant-workload cycling test in the SD group (*p* = 0.008), but not in the CON group (*p* = 0.515). Main pairwise comparisons in the SD group revealed a significantly more negative valence on day 2 vs day 1 (*p* = 0.013), a significantly more positive valence on day 3 vs day 2 (*p* = 0.032), and no significant difference in affect between day 1 and day 3 (*p* = 1.000).

HR showed a significant group × day × time interaction effect (*p* = 0.047) (Fig. 3C). The follow-up two-way ANOVAs performed at each time point (5 to 40 min) during the 40-min constant-workload cycling revealed a group × day interaction effect at the 5-min time point (*p* = 0.043). The follow-up one-way ANOVAs showed only a tendency towards a significant HR decrement in the CON group (*p* = 0.080).

No significant group × day × time (*p* = 0.070) and group × day (*p* = 0.308) interaction effects were observed for blood lactate (Fig. 3D).

### 20-min time trial—perceptual and physiological responses

**Fig. 4:**
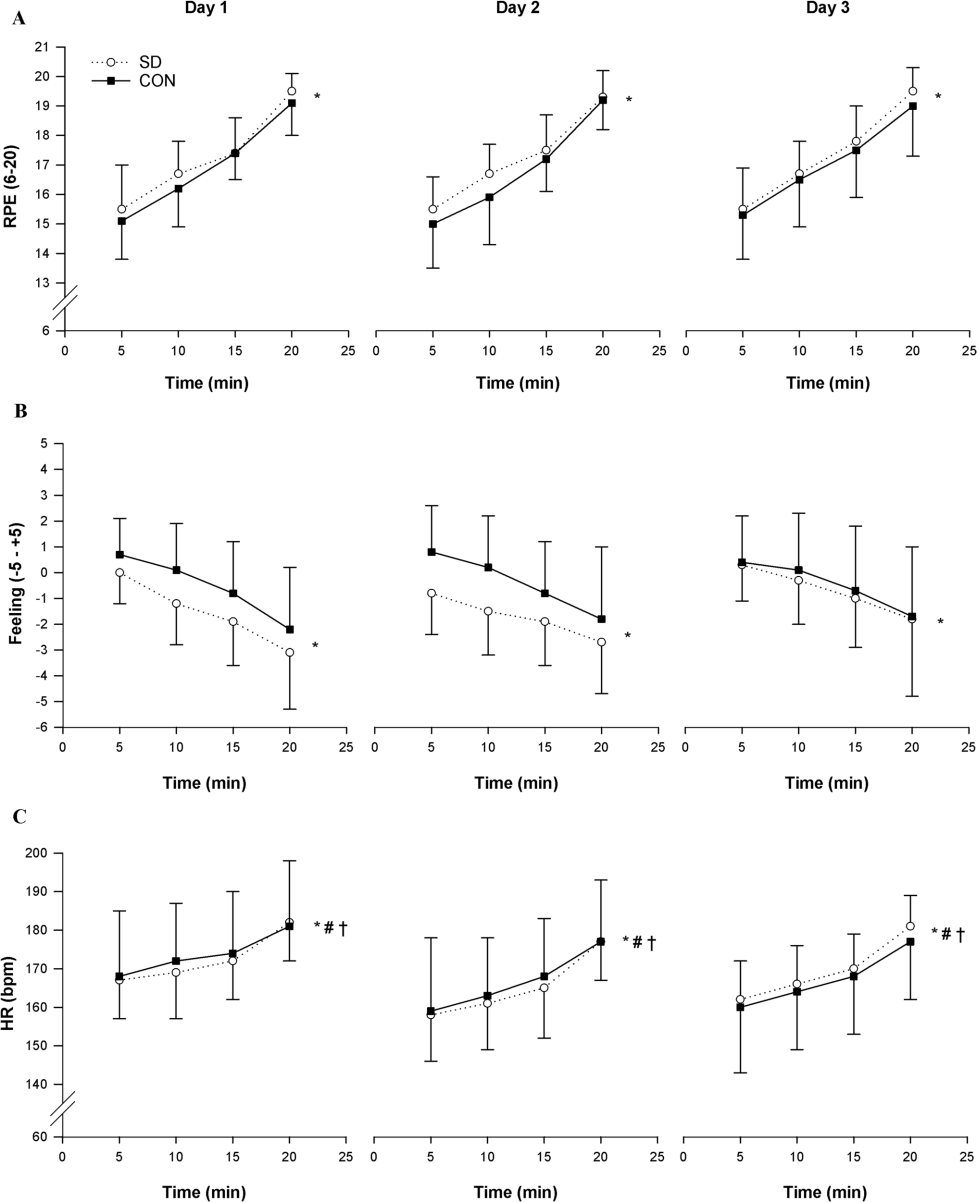
Group mean ± SD changes in RPE (**A**), Feeling scale (**B**), and HR (**C**) measured during the 20-min time trial on day 1, day 2, and day 3 in the SD group (open line) and CON group (solid line). * = significant main effect of time (*p* < 0.001); # = significant main effect of day (*p* < 0.001); † = significant group × day interaction effect (*p* < 0.05); ¶ = significant day × time interaction effect (*p* < 0.01)

Significant main effects of time were observed for RPE, affective valence, and HR (*p* < 0.001) (Fig. 4). No significant interaction effects were found for RPE (Fig. 4A) and affective valence (Fig. 4B) during the 20-min time trial. However, a significant group × day interaction effect (*p* = 0.027) was found for HR (Fig. 4C). Follow-up analysis revealed lower HR during the 20-min time trial in the SD group on day 2 (*p* < 0.001) and day 3 (*p* = 0.042) vs day 1, but higher HR at day 3 vs day 2 (*p* = 0.005). Similarly, the CON group showed lower HR compared to baseline (day 1 vs day 2, *p* = 0.018; day 1 vs day 3, *p* = 0.001), but no significantly higher HR at day 3 vs day 2 (*p* = 0.320).

**Fig. 5:**
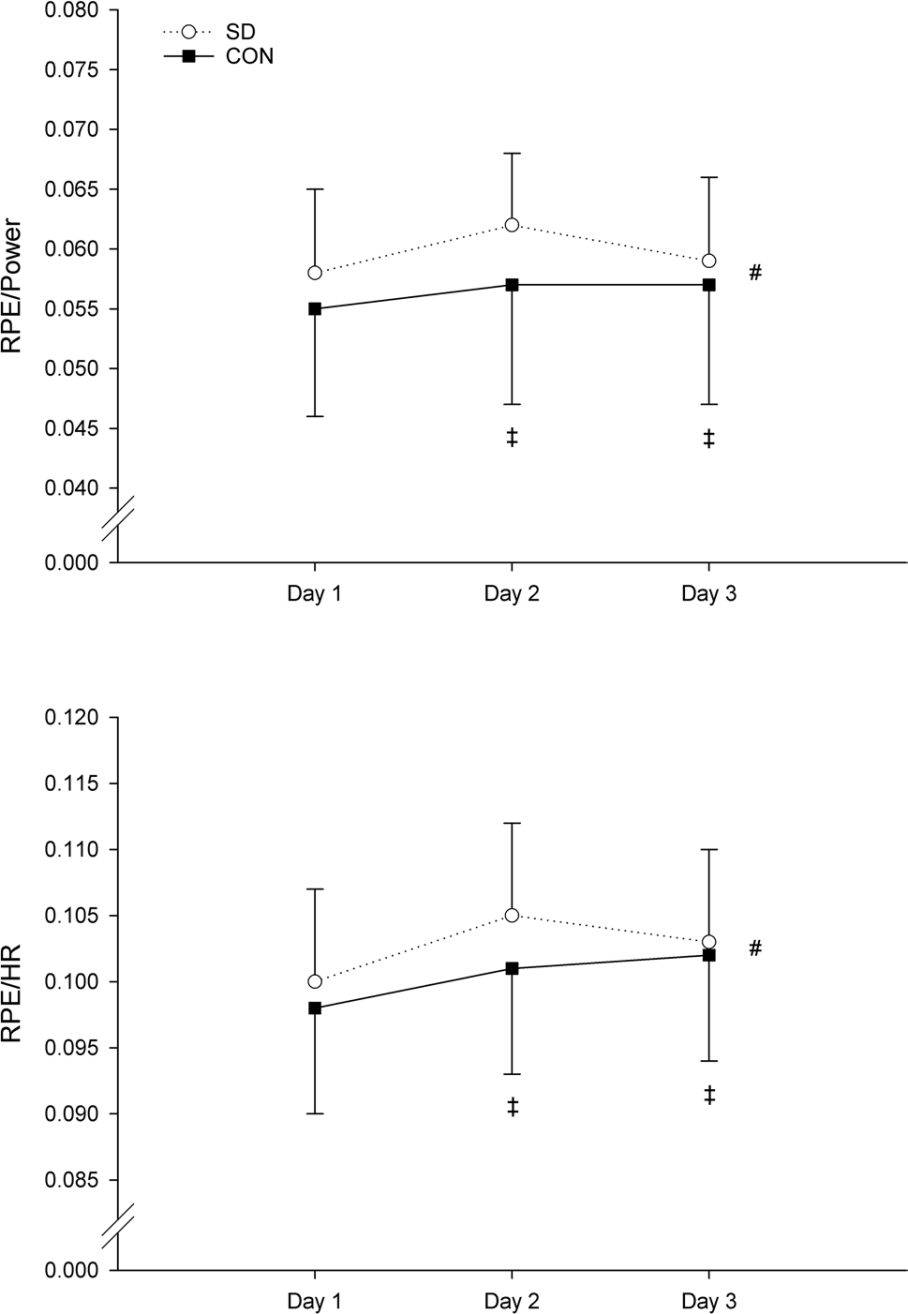
Grand mean ± SD RPE/Power and RPE/HR during the 20-min time trial on day 1, day 2, and day 3 in the SD group (open line) and CON group (solid line). # = significant main effect of day (*p* < 0.01); ‡ = significant main pairwise comparison of day 2 compared to day1 (*p* < 0.01), of day 1 compared to day 3 (RPE/HR only, *p* < 0.01), and of day 2 compared to day 3 (RPE/power only, *p* < 0.05)

During the 20-min time trial, RPE/power (*p* = 0.208) and RPE/HR (*p* = 0.161) ratios revealed no significant group × day interaction effects. However, a significant main effect of day was found for RPE/power (*p* = 0.007) and RPE/HR (*p* = 0.002). Pairwise comparisons across days showed significant higher RPE/power (*p* = 0.003) and RPE/HR (*p* = 0.003) ratios at day 2 compared to day 1, higher RPE/HR ratio (*p* = 0.005) at day 3 compared to day 1, and lower RPE/power ratio (*p* = 0.047) at day 3 compared to day 2 (Fig. 5).

Blood lactate production (i.e., the difference between blood lactate at the end of the 20-min time trial and at the end of the 40-min constant-workload cycling) did not show any significant main effect of day (*p* = 0.278) or interaction effect (*p* = 0.810).

### Moderate-intensity and high-intensity endurance cycling performance

**Fig. 6:**
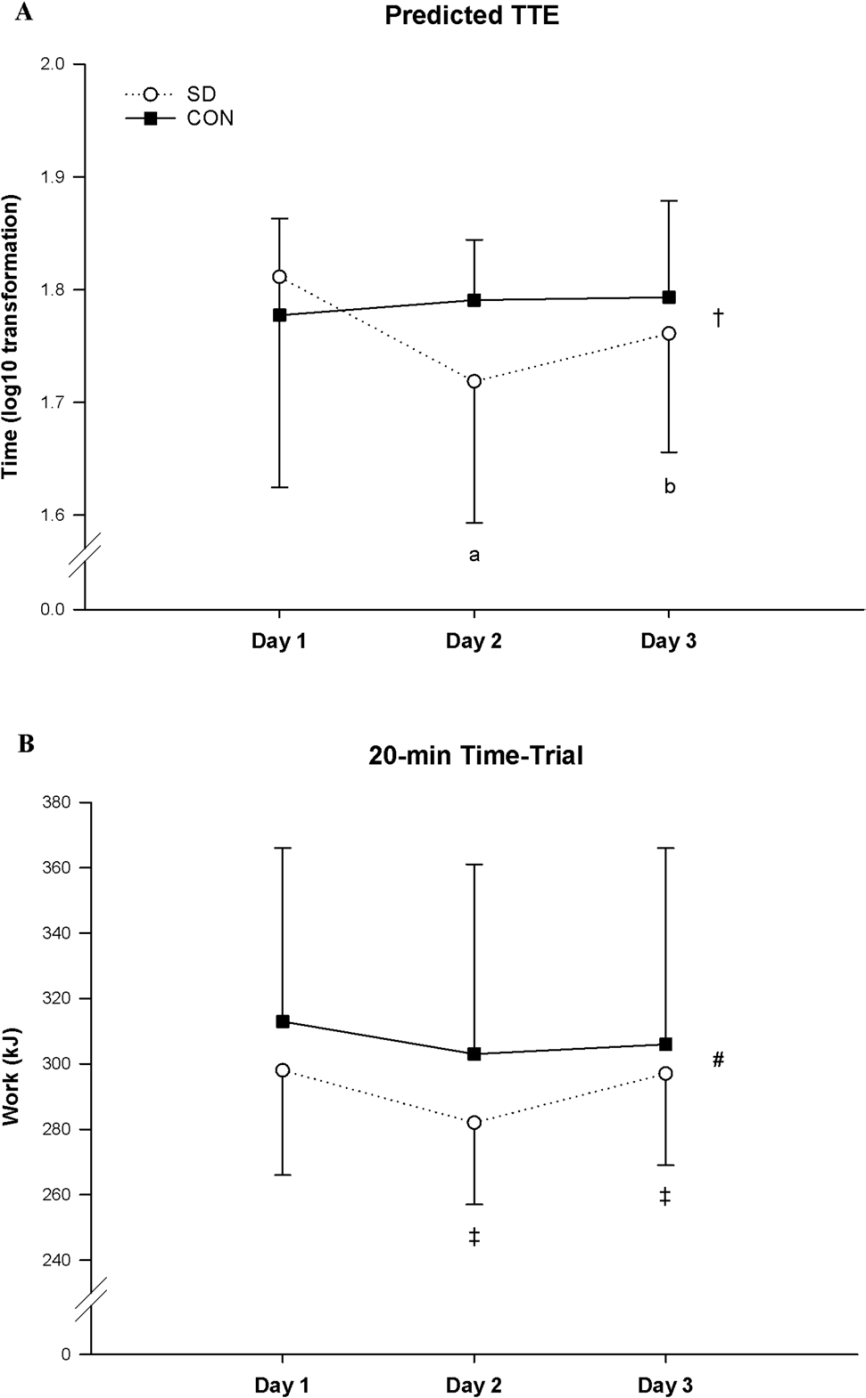
Group mean ± SD Log10 predicted TTE and work variations measured during the 40-min constant-workload cycling and 20-min time trial on day 1, day 2, and day 3 in SD (open line) and CON group (solid line). † = significant group × day interaction effect (*p* < 0.05); a = SD group different from day 1 (*p* < 0.05); b = SD group different from day 2 (*p* < 0.05); # = significant main effect of day (*p* < 0.001); ‡ = significant main pairwise comparison of day 2 compared to day1 (*p* < 0.001) and day 3 compared to day 2 (*p* < 0.01)

A significant group × day interaction effect was observed for moderate-intensity endurance cycling performance (*p* = 0.035) (Fig. 6A and Table 1). Results from the one-way ANOVA revealed a significant decline in predicted TTE on day 2 (day 1: 1.81 ± 0.19 log10(min); day 2: 1.72 ± 0.13 log10(min), *p* = 0.027) and a subsequent improvement on day 3 (day 3: 1.76 ± 0.11 log10(min), *p* = 0.033) in the SD group. No significant differences were observed between day 1 and day 3 (*p* = 0.223). No significant changes were found in the CON group (*p* = 0.613).

**Table 1:** Predicted TTE per group on day 1, day 2, and day 3

	Day 1 (min)	Day 2 (min)	Day 3 (min)
SD group (*N* = 13)	56 (33)	49 (26)^a^	57 (21)^b^
CON group (*N* = 13)	60 (18)	65 (10)	63 (14)

Data are shown as medians (interquartile ranges)

*P* values refer to ANOVAs conducted on normalized data (see text for more details)

^a^*vs* day 1 *p* < 0.05

^b^*vs* day 2

No significant group × day interaction effect was observed on high-intensity endurance cycling performance. (*p* = 0.126) (Fig. 6B). However, a significant main effect of day was found (*p* < 0.001). Subsequent main pairwise comparisons across days showed a significant decline in work during the 20-min time trial on day 2 (day 1: 305.9 ± 43.7 kJ, day 2: 292.5 ± 45.1 kJ, *p* < 0.001) and a subsequent improvement on day 3 compared to day 2 (day 3: 301.8 ± 46.1 kJ, *p* = 0.003). No significant differences were observed between day 1 and day 3 (*p* = 0.219).

## Discussion

The main findings of this study revealed that: (1) a 25-h SD has a negative impact on sleepiness and vigilance and a following night of recovery sleep restores them to baseline; (2) 25-h SD appears to negatively affect moderate-intensity endurance cycling performance, but one-night recovery sleep seems sufficient to restore performance to baseline levels; (3) 25-h SD does not appear to have a detrimental effect on high-intensity endurance cycling performance in the context of 3 consecutive days of testing.

### Markers of SD and recovery sleep

In agreement with previous studies (Dinges et al. 1997; Belenky et al. 2003), our findings showed an increase in subjective levels of sleepiness after 25-h SD followed by a subsequent decrease following one-night recovery sleep.

From a neurophysiological perspective, the AAT revealed a decrease in the mean alpha power in the frontal cortex following SD. These findings confirm a reduction in alertness levels on day 2 in the SD group, and a subsequent return to baseline levels on day 3 following recovery sleep.

As expected, 25-h SD impaired PVT performance by increasing simple reaction time and number of lapses in the SD group. Moreover, the subsequent night of recovery sleep proved beneficial in restoring performance to baseline levels. These results align with previous studies demonstrating clear decrements in vigilance and sustained attention following SD (Doran et al. 2001; Van Dongen et al. 2003; Adam et al. 2006; Lim and Dinges 2008), and a rapid recovery to well-rested performance levels after only one night of normal sleep (Adam et al. 2006).

### Effects of SD and recovery sleep on moderate-intensity endurance cycling performance

While no alterations in HR and blood lactate concentrations were found during the 40-min constant-workload cycling, RPE and affect were negatively affected by 25-h SD. These findings align with previous experiments (Martin 1981; Martin and Gaddis 1981; Martin and Haney 1982; Bond et al. 1986; Plyley et al. 1987; Symons et al. 1988; Mougin et al. 1989, 1991; Azboy and Kaygisiz 2009; Oliver et al. 2009; Temesi et al. 2013), and affirm the detrimental impact of acute SD on perceptual responses during sub-maximal endurance exercise. In addition to that, the TTE predicted using RPE showed that 25-h SD would have negatively affected moderate-intensity endurance cycling performance and one-night recovery sleep would have been sufficient to restore performance to baseline.

According to the psychobiological model of endurance performance (Marcora 2008), perception of effort and potential motivation (defined as the maximum effort a person is willing to exert to satisfy a motive (Wright 2008)) are the main limiting factors of endurance performance. In the present study, even though a lower intrinsic interest in the cycling task was reported on day 2 following SD compared to day 1 and 3, participants’ motivation to succeed in the cycling task, which is essential for high performance achievements (Matthews et al. 2001), was preserved. The absence of changes in task success motivation during our experiment corroborates the assumption that perception of effort could have been the key factor affecting moderate-intensity endurance performance during SD and following recovery sleep (Temesi et al. 2013).

To the best of our knowledge, this is the first study investigating SD and recovery sleep in the context of 3 consecutive days of exercise testing. A similar experimental design was used to test the effects of SD and following recovery sleep during a cycling all-out TTE, showing no differences in performance (Hill et al. 1994). However, because of its short duration (i.e., less than 75 s), the exercise task chosen cannot be defined as an endurance type of exercise (Gastin 2001), and therefore it is not comparable with our results. Moreover, the presence of no control condition/group might have limited the findings of that experiment. Further research is essential to better understand the role played by perception of effort on moderate-intensity endurance performance during SD and recovery sleep states and to investigate the mechanisms underlying our findings.

### Effects of SD and recovery sleep on high-intensity endurance cycling performance

Performance during the 20-min time-trial was not impaired by 25-h SD. The results highlight the same performance pattern between the two groups over the 3 experimental days. Nevertheless, a main pairwise comparison across days revealed a general performance decline on day 2 and a subsequent improvement on day 3. Several reasons might explain the current results.

The first and perhaps most plausible explanation is that 25-h SD does not impair high-intensity endurance performance. The different impact of acute SD on moderate vs high-intensity endurance performance found in our study may be explained by the effect of exercise intensity on attentional focus (Hutchinson and Tenenbaum 2007). At high intensity, perception of effort and exercise tolerance seems more influenced by the overwhelming physiological sensations (e.g., leg effort and dyspnea) that dominate focal awareness. On the contrary, at moderate intensity, participants may have been more affected by feelings of fatigue and sleepiness associated with SD. Our findings that SD affects moderate-intensity endurance performance more than high-intensity endurance performance are confirmed by a recent meta-analysis which showed that endurance exercise lasting more than 30 min is more affected by SD than endurance exercise lasting less than 30 min (Lopes et al. 2023).

It is important to highlight though that our results are in contrast with what was previously found during a 30-min running time trial after 30-h SD (Oliver et al. 2009). These results suggest that acute SD may also have a different impact based on the type of activity performed. In particular, cycling might be less sensitive to SD-induced impairments, due to its general lower level of fatigability. Indeed, for exercise bouts of the same intensity and duration, cycling reaches lower levels of central fatigue and induces less muscle damage than running (Lepers et al. 2000, 2002; Millet et al. 2002, 2003). Cycling on a stationary ergometer is also likely to be less demanding from a neurocognitive perspective due to lower balance and motor control requirements. These factors may explain why SD negatively affects a 30-min running time trial and not a 20-min stationary cycling time trial. Future research needs to be conducted to investigate these hypotheses.

Another important aspect to consider is related to subjects’ sensitivity to SD. There is considerable inter-variability among physically active people on the effects of acute SD on endurance exercise performance, with individual performance changes varying from + 4% to -40% following SD (Martin 1981; Oliver et al. 2009). Therefore, it could be the case that the participants recruited in the present study were not sufficiently vulnerable to SD.

Lastly, the type of experimental design used might also have influenced the findings of this study. Due to our interest in investigating the effects of acute recovery sleep on endurance exercise performance, subjects were required to perform the same cycling task three times, in 3 consecutive days. This, along with the fact that participants were informed about their group allocation since the familiarization visit (due to participants’ organizational reasons), might have influenced the performance pattern of the CON group over the 3 days of testing. Indeed, it seems that the CON group followed the same pattern of the SD group, particularly on day 2, with a 3.2% drop in performance, versus a 5.4% in the SD group. Considering that SD was demonstrated not to affect pacing during time trials (Oliver et al. 2009), and that no performance decrements have been observed in the context of consecutive days of cycling time trials in rested cyclists (Hickey et al. 1992; Feli et al. 2006), we believe that the CON group did not underperform on day 2 due to fatigue-related factors, but likely adopted a pacing strategy, knowing that they would have to perform again the following day. Moreover, they may also have been influenced by the so-called “last ride effect” on day 3 (Hickey et al. 1992). This pacing strategy over the 3 days of endurance exercise may have masked the negative effect of SD on a self-paced test like the 20-min time trial.

### Limitations

This study has some limitations that should be acknowledged. First, we did not perform an a priori sample size estimation analysis due to the lack of randomized controlled trials on sleep deprivation and recovery sleep effects on endurance exercise performance, and the predominance of cross-over designs in prior research, which challenged the conduction of a power analysis. As this is the first study using this type of design in the field, a convenience sample was used, though its size is comparable to previous studies.

Second, only male participants took part in this study. Although the study was open to both male and female participants, no female cyclists or triathletes volunteered. Therefore, future research is warranted to investigate the effects of sleep deprivation and recovery sleep on endurance cycling performance in women.

## Conclusion

In conclusion, 25 h of SD impaired moderate-intensity endurance cycling performance through higher perception of effort and one subsequent night of recovery sleep was sufficient to bring performance back to resting levels. On the contrary, high-intensity endurance cycling performance was not affected by 25 h of SD. To the best of our knowledge, this is the first randomized-controlled trial testing acute SD and subsequent recovery sleep over 3 consecutive days of exercise testing and further research is, therefore, needed. From a practical perspective, the objective quantification of a recovery sleep effect is essential in the field of sport and exercise and can be translated into evidence-based strategies for trainers and athletes interested in reducing the negative effects of lack of sleep during training and multi-day endurance and ultra-endurance competitions.

## Data Availability

The datasets generated during and/or analysed during the current study are available from the corresponding author on reasonable request.

## Abbreviations

AAT: Alpha Attenuation Test
ANOVA: Analysis of variance
BRUMS: Brunel Mood Scale
CON: Control
EEG: Electroencephalography
HR: Heart rate
MEQ: Morningness-Eveningness Questionnaire
PSQI: Pittsburgh Sleep Quality Index
PVT: Psychomotor vigilance task
RPE: Ratings of perceived exertion
RT: Reaction time
SD: Sleep deprivation
SD: Standard deviation
TTE: Time to exhaustion
$\dot{\mathrm{V}}{\mathrm{O}}$_2peak_: Peak oxygen uptake
