# Correction: The effects of one-night sleep deprivation and one-night recovery sleep on endurance cycling performance

**DOI:** 10.1007/s00421-025-06087-4

**Published:** 2026-02-17

**Authors:** Chiara Gattoni, Michele Girardi, Amir-Homayoun Javadi, Barry Vincent O’Neill, Samuele Maria Marcora

**Affiliations:** 1 The Lundquist Institute for Biomedical Innovation at Harbor-UCLA Medical Center, Torrance, CA USA; 2 School of Psychology, University of Kent, Canterbury, UK; 3 Unilever Science & Technology, Unilever R&D Colworth Science Park, Sharnbrook, Bedford, UK; 4 Department of Quality of Life Studies, University of Bologna, Rimini, Italy; 5 School of Sport and Exercise Sciences, University of Kent, Canterbury, UK

**Correction: European Journal of Applied Physiology (2025)** 10.1007/s00421-025-05908-w

In the original version of this article, oxygen uptake was updated incorrectly as $\dot{\mathrm{V}}$2peak instead of $\dot{\mathrm{V}}$O2peak in the abstract, abbreviations and main text. 

The original article has been corrected.

